# Functional Neurological Disorder Management in a Pediatric Hospital: A Retrospective Chart Review

**DOI:** 10.1155/ijpe/4549313

**Published:** 2026-10-07

**Authors:** Mateya Rettig, Nicole Sweetland, Yanhao Shao, Laurie Thompson, Laurey Brown, Meredith Norwood

**Affiliations:** ^1^ Northwestern University–Chicago Campus Chicago Illinois USA; ^2^ Ann and Robert Lurie Children′s Hospital of Chicago Chicago Illinois USA; ^3^ The George Washington University Washington District of Columbia USA gwu.edu

**Keywords:** functional neurological disorder, neurology, pediatric, psychiatry, quality improvement, rehabilitation

## Abstract

**Background:**

Functional neurological disorder (FND) is a complex, multinetwork brain disorder that is characterized by the presence of a broad range of neurological symptoms in the absence of neurological disease or damage. For pediatric patients requiring an in‐patient stay, misdiagnosis, lack of provider consistency and diagnostic education, frequent testing and work‐up, and invasive procedures are often reported. This review will characterize the population, identify current state for receiving diagnosis, detect variability in service delivery, and provide information to evaluate use of resources.

**Methods:**

This retrospective chart review examined electronic medical records (EMRs) of 59 patients at a pediatric teaching hospital who were admitted following a visit to the emergency department. Records were compiled by the rehabilitation department with inclusion criteria as having a documented diagnosis of FND or as having a similar functional movement related diagnosis such as chorea, panic disorder, and psychogenic nonepileptic seizure (PNES). Extracted data included the following: demographics, patient characteristics, admission characteristics and experience, and discharge recommendations.

**Results:**

Fifty‐nine charts were initially reviewed with 57 meeting inclusion criteria. The subjects in this study had a mean age of 13.7 years (SD: 6.33) and 80.7% were female. The most commonly reported psychiatric diagnoses included anxiety (66.7%), ADHD (29.6%), and depression (22.2%). The most commonly reported FND symptoms at admission were weakness (47.4%), gait instability (28.1%), sensory disturbances (26.3%), and tremor (19.1%). Physical therapy consultations were the most frequently performed (66.7%), followed by occupational therapy (52.6%), psychiatry (38.6%), and psychology (5.3%). No interdisciplinary care conferences were performed. The mean length of stay was 5.13 days (SD: 4.45 days). Readmission rate after 30 days was 16.5%.

**Conclusions:**

This retrospective EMR review identifies opportunities to improve care for pediatric FND, including reducing unnecessary imaging, testing, and readmissions through strengthened provider, patient, and family education. Findings also highlight inconsistent referrals to physical therapy, occupational therapy, and mental health services. Addressing these gaps may enhance patient experience, support more coordinated interdisciplinary care, and reduce healthcare utilization for this under‐recognized population.

Key Points

• Pediatric patients with functional neurological disorder in inpatient settings often experience a lack of provider consistency, inadequate diagnostic education, frequent testing, and misdiagnosis
• Despite the known benefit of mental health services for patients with FND, very few participants received mental health services during their inpatient stay
• Opportunities exist for improved consistency in referrals for PT, OT, and mental health services
• Emphasizing a multidisciplinary care approach and promoting provider education can improve the lived experiences of individuals with FND

## 1. Introduction

Functional neurological disorder (FND) is a complex, multinetwork brain disorder that is characterized by the presence of a broad range of neurological symptoms in the absence of neurological disease or damage [1]. Functional symptoms are highly variable but may include gait instability, change or loss of vision, tremor, seizure‐like symptoms, numbness, weakness, pain, discoordination, ataxia, and difficulties with swallowing, among others (DSM‐5 [2]; [3]). Evidence suggests that approximately 2–3 patients per week are presenting to high volume tertiary pediatric emergency departments (EDs) with functional neurological symptoms [4]. Data for pediatric FND incidence is limited and varying in clinical significance, with recent reports of 6–18 per 100,000 children per year [5].

The clinical experience of individuals with FND is frequently complicated and challenging, with the pediatric trajectory remaining relatively underdocumented in current literature. Current literature estimates that in the United States, FND accounts for 1264 pediatric admissions among 965,132 total hospitalizations (~0.13 per 1000) and 1% of all pediatric neurology admissions [5]. For these patients there is often a storied path toward a diagnosis with evidence for misdiagnosis, lack of provider consistency and messaging, frequent testing and work‐up, and invasive procedures. This leads to an estimated inpatient care cost of 75 million dollars annually, with total cost per admission rising faster than other neurological disorders [6]. Although recently significant work has been done to improve the standard of care and establish a standardized approach when diagnosing and treating these individuals, there is still progress to be made. Enlightening the qualitative experience with quantitative information will assist in driving forward ongoing change.

The study complements a previous publication by Brown et. al., [7] which outlined the process and experience at one institution for creating a patient care pathway that improves patient flow and increases equity among patient groups. This pathway also identified knowledge gaps and attempted to provide different educational resources for providers as well as patients and caregivers. It highlighted a team approach for establishing consensus and creating feasible and positive change. This present study provides a retrospective chart review that quantitatively enlightens the background that led to the pathway′s creation. It seeks to characterize the population, identify the current state for receiving the diagnosis, detect variability in service delivery and evaluate use of resources in the real world. It further assists in identifying where improvement is needed, and where qualitative change may focus in the future. Previous studies have utilized a retrospective chart review in a similar manner. This study is aimed at strengthening and echoing these concerns while bringing to light an even more expansive picture for FND [8].

## 2. Methods

### 2.1. Setting

This is a retrospective electronic medical record (EMR) review conducted at a 312‐bed pediatric teaching hospital in a large urban area. The data review for this study began in November 2024 and ended in March 2025.

## 3. Retrospective Chart Review

A retrospective EMR review was conducted on a sample of 59 patients who were admitted to a pediatric teaching hospital following a visit to the ED. The patient list for review was compiled by the rehabilitation department from March 2012 to January 2025 with inclusion criteria based on primary presentation for movement disruption and subsequent referral for rehabilitation services including physical or occupational therapy (OT). Some of these patients had a documented diagnosis of FND (64.9%) whereas others (35.1%) had related primary diagnoses such as functional chorea, panic disorder, or psychogenic nonepileptic seizures (PNES) which included a functional movement component. Although FND was not listed as a diagnosis in 100% of cases, all charts reviewed and included in the list demonstrated a disordered functional movement that was not otherwise specified, thus meeting inclusion criteria for the patient list. The sample of patients was admitted between March 1, 2012, and January 21, 2025. All data from patient charts were deidentified and assigned a study number. Protected health information was not reused or disclosed. Data extraction was performed by trained research personnel and utilized a data abstraction form in REDCap, a secure web application for building and managing online surveys and databases. Data extracted included: age at time of service, date of birth, sex, gender identity, insurance provider, zip code, race, ethnicity, if FND was documented, co‐occurring medical diagnoses, psychiatric diagnoses, history of self‐harm, admitting diagnoses, duration of symptoms at admission, FND symptom descriptors, imaging and testing performed, consultations performed while inpatient, date of consultations, discharge recommendations/plan, discharge date, length of stay, 30‐day readmission, discharge diagnoses, future services scheduled at the identified hospital, and services that the patient was already being seen by. Data extraction was based on clinical documentation with the potential for multiple diagnoses assigned to a single patient.

Study protocol was received and approved by an IRB (2023‐6250) and was declared as exempt under the post‐2018 rule. The research was conducted according to the Declaration of Helsinki.

### 3.1. Data Analysis

REDCap software was used to assess the standard deviation and frequency of the nominal variables.

## 4. Results

Initially, 59 patient charts were reviewed as a component of the available patient list created by rehabilitation services. Two charts were excluded because of missing discharge summaries and services primarily conducted at outside hospitals.

### 4.1. Demographics

A total of 57 subjects participated in the study with a mean age 13.7 years (SD: 6.33). Among these, 19.3% were male, and 80.7% were female. A total of 54.4% of the subjects identified as women, 14% identified as men, and 31.6% in which patient gender identity preference was labeled as not indicated (Table 1). The majority of participants were from the city and area surrounding the hospital. The population consisted of 40.4% White, 31.6% Black or African American, 8.8% Asian, 1.8% American Indian, 1.8% Native Hawaiian or other Pacific Islander, and 17.5% other. A total of 21.1% of subjects were Hispanic or Latino or of Spanish origin, and 78.9% were not Hispanic, Latino, or of Spanish origin.

**Table 1:** Demographics.

Category	Variable	Value
Sample	Total participants	57
	Mean age	13.7 years (SD 6.33)

Sex assigned at birth	Male	19.3%
	Female	80.7%

Gender identity	Women	54.4%
	Men	14.0%
	Not indicated	31.6%

Race	White	40.4%
	American Indian	1.8%
	Asian	8.8%
	Black or African American	31.6%
	Native Hawaiian or Pacific Islander	1.8%
	Other	17.5%

Ethnicity	Hispanic/Latino/Spanish origin	21.1%
	Not Hispanic/Latino/Spanish origin	78.9%

Geographic origin	Residence	Majority from city/area surrounding hospital

Co‐occurring medical diagnoses	Seizures	22.9%
	Abdominal pain	18.8%
	Headaches	14.6%
	Syncope	14.6%
	Epilepsy	2.1%
	POTS	10.4%
	Chronic pain	12.5%
	Asthma	10.4%
	Migraine	12.5%
	Arthritis	4.2%
	Dizziness	8.3%
	Nausea	10.4%
	Tourette′s syndrome	4.2%
	Concussion	4.2%
	Sensory processing disorder	2.1%
	Long COVID	0%
	Chronic Lyme disease	0%

### 4.2. Patient Characteristics

There were a number of co‐occurring medical diagnoses during hospital stay. The most commonly reported were seizures (22.9%), abdominal pain (18.8%), headaches (14.6%), and syncope (14.6%). Additional co‐occurring medical diagnoses included epilepsy (2.1%), postural orthostatic tachycardia syndrome (POTS) (10.4%), chronic pain (12.5%), asthma (10.4%), migraine (12.5%), arthritis (4.2%), dizziness (8.3%), nausea (10.4%), Tourette′s syndrome (4.2%), concussion (4.2%), and sensory processing (2.1%). No patients had long COVID or chronic Lyme disease, but these diagnoses were considered, specifically (Table 1). Other diagnoses were listed in 58.3% of cases and were not specifically collected. Anxiety was the most frequently reported psychiatric diagnosis, affecting 66.7% of participants. ADHD was reported in 29.6% of participants and depression in 22.2%. Additional reported psychiatric diagnoses included PTSD/trauma (14.8%), developmental delay (3.7%), autism (14.8%), and disruptive behavior disorder (14.8%). No patients had a diagnosed learning disability or documented adjustment disorder. Other diagnoses were listed in 18.5% of cases and were not specifically collected. Additionally, 12.3% patients had a history of self‐harm.

Physical therapy (PT) was the most commonly utilized service preadmission, reported by 63.6% of participants. OT was received by 36.4%, and 18.2% were engaged in mental health services (Table 1). Additionally, 27.3% reported receiving other types of services preadmission that were not specified in the predefined categories.

### 4.3. Admission Characteristics and Experience

The most commonly reported FND symptom at admission included weakness (47.4%) and gait instability (28.1%). Sensory disturbances were noted in 26.3% of patients, whereas tremor was observed in 19.1%. Seizures were reported by 17.5% of patients, and syncope occurred in 15.8% of patients. Other symptoms included blindness (3.5%), visual disruption (7.0%), swallowing difficulties (5.3%), and speech issues (10.5%) (Figure 1).

**Figure 1:**
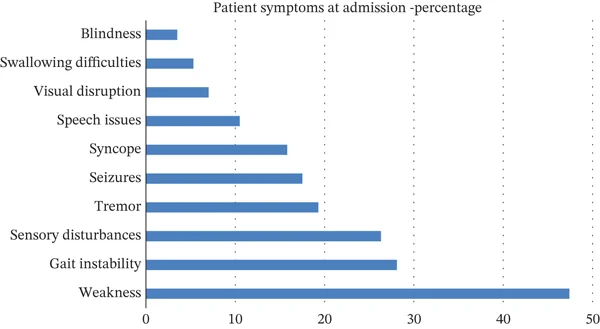
Patient symptoms at admission–percentage.

Of the 57 subjects, imaging and tests performed included MRI (49.1%), CT scans (24.6%), and x‐rays (26.3%). Electroencephalograms (EEGs) were conducted in 7.0% of patients, whereas video EEG (vEEG) was performed for 10.5% of patients. No patients underwent lumbar puncture. Other tests were conducted in 21.1% of patients, and 12.2% of patients did not have any imaging performed.

PT consultations were the most frequently performed, accounting for 66.7% of cases, followed by OT at 52.6%. Psychiatry consultations were conducted for 38.6% of participants, whereas psychology consultations were less common (5.3%). Additional consultations included pain (1.8%); Shirley Ryan Ability Lab (10.5%); gastrointestinal (3.5%); cardiac (3.5%); child life (1.8%); social work (8.8%) and orthopedics (1.8%) (Figure 2). None of the 57 patient cases had interdisciplinary care conferences performed.

**Figure 2:**
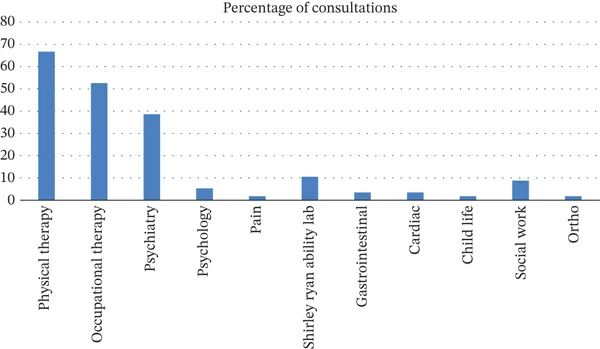
Consultations performed.

The mean length of stay was 5.13 days (SD: 4.45 days). Readmission rate after 30 days was 16.5%.

### 4.4. Discharge Recommendations

Of the 57 subjects, the discharge plans included: outpatient (OPT) PT (54.4%), OPT OT (26.3%), OPT psych (22.8%), day rehab (1.8%), inpatient psych (3.5%), inpatient rehab (8.8%), intensive pain program (7.0%), no services recommended (5.3%), PCP follow‐up (7.0%), and other (45.6%). Within the “other” category, the breakdown is as follows: cardiology (19.2%), neurology (26.9%), endocrinology (15.4%), and speech and language pathology (11.5%).

## 5. Discussion

The findings of this chart review enlighten the current state of admission for pediatric patients with movement presentations of FND in regard to patient demographics, characteristics, and admission and discharge details. This study was consistent with previous demographic data in that a majority of the patients were adolescent females [4, 9]. The data are consistent with existing literature in that there were high rates of co‐occurring diagnoses and psychological disturbances in the patient sample [9, 10]. Acknowledgement of these consistencies assists in describing the varying phenotypic presentations for these individuals, as well as identifying potential accompanying factors in the development of FND.

Our findings regarding admission experience were also consistent with previously cited literature with variable symptom presentation, frequent imaging, and length of stay ranging from 1 day to 5 months [8]. This speaks to a high utilization of healthcare dollars, as well as inconsistent patient experiences. We also observed a high number (~20%) of 30‐day readmission, which suggests poor symptom management at initial discharge and poorly established follow‐up care. It is difficult to shift from a culture of conceptualizing somatic action as solely psychological and outside the realm of physical medicine as FND has been known to sit at the intersection of neurology and psychology [3, 11]. As paradigms shift and knowledge of the characteristics, risk factors, and various presentations of FND improve, it is expected that this overutilization will improve as well [6, 12]. However, while “necessary” work‐up will always be indicated, what is considered necessary will hopefully be reduced through time.

Prior to admission, over half of the patients were already being followed by PT, whereas about one third of the patients were being seen by OT. Less than a quarter of the patients were being followed by mental health services prior to admission. Frequency of these services did not improve following admission to the hospital. There were also a number of patients who were experiencing speech concerns and swallowing disturbances, yet there were no referrals for speech therapy during the in‐patient stays or in any discharge plans.

These referral trends provide a disparate look at what is recommended versus the standard of care documents which support both PT and OT as hallmarks for improvement and referrals to speech therapy when that specific expertise is warranted [13–15]. There are likely many considerations driving this concern, some that have been previously addressed and some of which create future opportunities for intervention. This institution has established a local provider resource list for physical and OT in the attempt to eliminate the barrier for care delivery. However, this concern is more related to not being referred to therapies in the first place, which is a point of consideration for provider education. An additional consideration is the complementary and comprehensive role that PT and OT, together, are able to provide.

Given the essential role of ongoing mental health care for these individuals, we observe that while 38.6% received psychiatry referrals, very few (5.3%) received psychology services for care both during the hospital stay and at discharge [16, 17]. This indicates another area of need for provider education and suggests lack of knowledge about FND care within this setting as well as systemic influence for navigating and managing appropriate referrals. Psychology plays a pivotal role in advancing the understanding of the FND diagnosis and in providing therapeutic services that drive healing forward. Obtaining appropriate provider referrals in an efficient manner should be an area of significant consideration in moving the care continuum and practice forward. Additionally, there is a remarkable shortage of mental health treatment providers and an especially small percentage of providers that feel comfortable and confident in treatment of FND. Experienced therapists should provide consultation to existing providers, when appropriate to educate on the condition and adapt current CBT strategies.

These findings provide quantitative information of the current state for movement presentation of FND at this institution and enlighten the recommendations for the clinical care pathway that was created [7]. This work emphasizes that effective management of pediatric FND requires a coordinated, team‐based approach with clearly defined roles, consistent communication, and shared diagnostic frameworks. The pathway developed from this retrospective chart review reflects these principles: the need for early identification, streamlined diagnostic processes, and timely engagement of rehabilitation and mental health services. Our data highlight gaps that the optimized team model directly addresses—including inconsistent referral patterns, limited use of FND‐specific diagnostic codes, and variability in provider messaging. Integrating these insights into a structured clinical pathway has the potential to reduce unnecessary testing, improve continuity of care, and enhance provider confidence in managing FND. As previously noted, establishing a unified, interdisciplinary approach is foundational to improving outcomes, reducing healthcare utilization, and ensuring that pediatric patients receive timely, appropriate, and coordinated care across the continuum [7].

Future work should further the review and consider the length of stay and its associated costs and impact to the hospital system; analysis of racial and gender disparities and their impact on functional recovery; assessment of risks and past medical history that influences the symptoms associated with FND; opportunities for improving care access and strategies for reducing disparities and unnecessary treatment costs, healthcare utilization and improving timely access to diagnosis and treatment.

## 6. Limitations

Limitations to this study include a lack of information on the amount of time it took to receive a diagnosis of FND, which is important data to determine a benchmark for improvement. Additionally, patient lists were limited to individuals with movement‐based symptom presentations as these were the patients who had rehabilitation consultations performed. This limitation highlights a concern in that providers frequently documented these encounters under nonspecific diagnostic categories such as gait disturbance or muscle weakness, terms that encompass a broad range of neurological and nonneurological presentations. As a result, generating an accurate report of FND admissions was difficult, and the true scope of pediatric FND within the system is likely underestimated. This lack of diagnostic specificity complicates retrospective chart review, obscures clinical patterns, and reflects a broader issue in the field: inconsistent recognition and coding of FND across providers and settings. Improved provider education and standardized coding practices will be essential for strengthening future surveillance, quality improvement efforts, and research accuracy.

## 7. Conclusions

The results of this retrospective EMR review highlight meaningful opportunities to improve the care experience for pediatric patients with movement‐related FND. These findings contribute to the limited literature describing pediatric FND and illuminate the challenges in diagnostic pathways, inconsistent messaging, and substantial healthcare costs faced by this diagnosis. The review suggests opportunities for improvement including reduction of imaging, diagnostic testing, and readmission frequency through improved provider, patient, and family education about independent management strategies. Additionally, the data highlight a need for consistency in referrals for PT, OT, and psychology‐based health services—core interventions that remain unevenly applied despite their central role in functional recovery. It is anticipated that addressing these gaps will drive positive change for individuals with FND, reduce healthcare utilization, and support a more timely and coordinated interdisciplinary care.

**Nomenclature**
FND: functional neurological disorder
PT: physical therapy
OT: occupational therapy

## Funding

No funding was received for this manuscript.

## Ethics Statement

All data from patient charts were deidentified and assigned a study number. Protected health information was not reused or disclosed. Study protocol was received and approved by an IRB (2023‐6250) and was declared as exempt under the post‐2018 rule. The research was conducted according to the Declaration of Helsinki.

## Conflicts of Interest

The authors declare no conflicts of interest.

## Data Availability

The authors have nothing to report.
